# How is visual separation assessed? By counting distance units

**DOI:** 10.3389/fpsyg.2024.1410297

**Published:** 2024-05-30

**Authors:** Stephen Dopkins

**Affiliations:** Department of Psychological and Brain Sciences, George Washington University, Washington, DC, United States

**Keywords:** separation, position, distance perception, local sign, frontal plane, distance unit, size perception, counting distance units

## Abstract

How does the human visual system assess the separation between pairs of stimuli in a frontal plane? According to the *direct* (or subtractive) view the system finds the difference between the positions of the stimuli in a localization system. According to the *indirect* (or additive) view the system finds the number of instances of a distance unit lying between representations of the stimuli. Critically, position is explicitly represented under the direct view, with separation being derived from position. Position is not explicitly represented under the indirect view; separation is consequently inferred by counting an internal unit of distance. Recent results favor the indirect over the direct view of separation assessment. Dissociations between assessments of separation and position, various context effects in the assessment of separation, and suggestions that position information is not cleanly accessed argue against the direct view. At the same time, various context effects in separation assessment argue for the indirect view. Recent findings regarding the brain bases of vision are consistent with the indirect view. In short, recent results suggest that assessing the separation between two frontal stimuli involves integrating distance units between representations of the stimuli.

## Introduction

How does the human visual system assess the separation between pairs of stimuli in a frontal plane? Two sorts of explanation have been proposed (Watt, 1992). According to *direct* (or subtractive) accounts the visual system finds the difference between the positions of the stimuli in a localization system (Morgan and Regan, 1987; Burbeck and Yap, 1990; Stuart et al., 1993; Burbeck et al., 1996; Kohly and Regan, 2000). The localization system for separation assessment is sometimes understood in terms of local sign: Stimulation at a particular retinal position registers as a particular local sign. A particular local sign can be represented by a particular neural element (Matin, 1972; Rose, 1999) or a correlation among the patterns of activity in a set of neural elements (Koenderink, 1990, 2019). The separation between two stimuli can be derived from differences in local sign. For example, Burbeck and Hadden (1993) proposed that separation is assessed by a system of linked position encoders. When a pair of test stimuli is presented, multiple pairs of position encoders are activated, with the position encoders in each pair being linked by a pre-existent connection giving a particular separation. The separation between the test stimuli is derived from the relative degree of activation for position-encoder pairs associated with different separations (See also Burbeck and Pizer, 1995).

According to *indirect* (or additive) accounts the visual system assesses the separation between two stimuli by finding the number of instances of a distance unit lying between representations of the stimuli (Tsal and Shalev, 1996; McGraw and Whitaker, 1999; MacEvoy and Fitzpatrick, 2006). For example, Hisakata et al. (2016) proposed that separation is assessed in terms of neural elements that express local distance units. The separation between two stimuli is assessed by integrating the units that fall between representations of the stimuli.

To highlight the differences between the direct and indirect accounts of separation assessment, it is useful to compare the way the two accounts might explain a fundamental fact about separation assessment – that the refinement of separation discrimination decreases in proportion to the separations being discriminated (often called Weber’s law for separation) (Wolfe, 1923). Direct accounts have attributed the Weber-like relationship to decreases in the resolution of position information with increases in eccentricity (Levi et al., 1988) and separation (Burbeck and Hadden, 1993). Indirect accounts could attribute the relationship to increases in the numbers of integration errors with increases in the magnitude of the assessed separation.

## Supporting the indirect view

The present paper argues for the indirect view of separation assessment. In support of this view note, first, the evidence that has been reported against the direct view. Assessments of separation and position are often dissociated in ways difficult to explain if position information supports separation assessment. For example, although observers make errors in assessing the separation between the vertices of a Müller-Lyer figure, observers fail to make errors in indicating the positions of those vertices (Gillam and Chambers, 1985). In addition, a recent study found reports of remembered separations and positions to be dissociated in a way that is difficult to explain if separations are mentally represented in terms of positions. In the crucial task participants remembered the separations and positions of points on an axis. Although the to-be-remembered positions determined the to-be-remembered separations, memories for the two sorts of information were dissociated; memory for the separations was subject to bias and scaling effects from which memory for the positions was free (Dopkins and Collier, 2024).

Additional evidence against the direct view comes from various context effects in separation assessment. For example, the separation between pairs of test stimuli depends on the separation between each of the individual test stimuli and flanker stimuli. The test–test separation is assessed as relatively large when the test-flanker separation is smaller than the test–test separation and relatively small when the test-flanker separation is larger than the test–test separation (Hess and Badcock, 1995). Similarly, the separation between pairs of stimuli is assessed as smaller the smaller the sizes of the stimuli and the greater the spatial frequency of their background (McGraw et al., 2012). Contextual dependence such as this is difficult to square with a comparison of position information. Further evidence against the direct view comes from a task in which participants make judgments of horizontal or vertical separation with respect to pairs of dots that differ on horizontal and vertical axes. The precision of a given horizontal or vertical separation judgment decreases with increases in the vertical or horizontal separation, respectively, between the dots. By implication, participants cannot selectively access information about horizontal or vertical position. This is inconsistent with a simple direct account, under which the horizontal and vertical position information, giving horizontal and vertical separation, respectively, should be selectively accessible (Dopkins, 2005; Dopkins and Sargent, 2014; Dopkins and Hoyer, 2015, 2018).

Also incompatible with the direct view is evidence against the local sign idea. A number of factors can shift the perceived location of a stimulus away from the location given by its retinal position (Suzuki and Cavanagh, 1997; Whitney and Cavanagh, 2000; Zimmermann et al., 2013). Further, evidence has been advanced that position is represented implicitly rather than explicitly. Analysis of the correspondence between stimuli and their retinal images implies that the visual system represents differential spatial relations between stimulus aspects rather than the aspects themselves (Lappin and Craft, 2000). In addition, physiological data support the capacity of the visual system for the implicit representation of position; the responses of large-receptive-field cells in lateral intraparietal monkey cortex are consistent with such representation (Sereno and Lehky, 2011). And imaging data suggest the use of such representation. Attention increases receptive field size and position discriminability in the ventral stream, with the conjunction of these increases suggesting implicit representation of position (Kay et al., 2015).

At the same time, support has been accumulating for the indirect view of separation assessment. A number of context effects have been reported that are easier to square with the indirect than the direct view. In separation assessment, a pair of dots is evaluated as being closer together following perception of a dynamic random dot array than following perception of a blank field (Hisakata et al., 2016). The effect only occurs when the dot pair is close to the array (Jovanovic et al., 2022), where the closeness of the dot pair to the array can be assessed in world-centered as well as retinotopic terms. Similar results have been observed as a function of motor adaptation (as a byproduct of tapping) (Petrizzo et al., 2020). To explain these results researchers have proposed that separation assessment involves integrating distance units along the path separating representations of the test dots and that the adaptation process increases the size of the distance unit such that the path for a given test-dot pair encompasses fewer instances of that unit.

Similarly, a pair of dots is evaluated as being closer following short (120 s) bouts of coordinated two-point stimulation at eccentricities that are optimal and values of separation that match rather than mismatch those of the test dots. The researchers who observed these results attributed them to the strengthening of lateral connections between distance units in V1, with the number of units between the representations of two points being the basis of the assessed separation between the points (Song et al., 2017). Such strengthening was suggested as a possible mechanism underlying the effects that Hisakata et al. (2016) observed.

The forgoing effects of adaptation and learning on accuracy were not associated with changes in precision. Consistent with this, Chambers et al. (2018) showed that adaptation to a textured annulus (a stimulus similar to those producing the forgoing effects of adaptation and learning) produces perceived compression in the region surrounded by the annulus but no change in crowding within that region. More recently, however, changes in precision have been observed that may reflect similar mechanisms as do the forgoing adaptation and learning effects. Precision in discriminating the mean separation from smaller and larger members in a critical set of separations was higher in a group of participants for whom additional levels of separation were interleaved between the critical levels. The critical separation levels, the average separation level, and the range of separation levels were held constant across the Interleaved group and a control group for whom additional separation levels were not interleaved (Dopkins and McIntire, 2022). Building on the account of Hisakata et al. the researchers proposed (1) that the separation between two points is assessed by integrating the distance units between representations of the two points, and (2) that the size of the distance units decreases as the separation levels tested are more closely spaced. The researchers noted the difficulty of explaining their results under a direct account, asking why, under such an account, assessing the separation between a pair of points should depend on the assessment of separation levels interleaved between the separation level of the points? A rival account of these results, however, is that the interleaved levels allowed more precise learning of the response criterion. In argument against this rival account, a similar effect of interleaved levels was observed in a follow-up task that required discriminating each of the separation levels tested in the original experiment from several smaller and larger levels. Because the Interleaved group in this follow-up task had to learn more response criteria than the Control group, the higher precision in the Interleaved group probably did not reflect better criterion learning (Dopkins, 2024).

Further support for the indirect view has come from tasks requiring assessment of vertical and horizontal separation (Dopkins and Galyer, 2020). Pairs of points have for years been known to be assessed as further apart when oriented on the vertical as opposed to the horizontal axis. The precision of vertical and horizontal separation assessment has not been examined, however. Using a new technique in which vertical and horizontal assessments are both made against the same implicit standard, Dopkins and Galyer demonstrated that, when the two sorts of assessment are made in the same context, vertical assessments are more precise than horizontal assessments. Noting the difficulty of explaining their results under a direct account Dopkins and Galyer proposed (1) that the separation between two points is assessed by integrating the distance units between the two points, and (2) that, when vertical and horizontal separation are assessed in the same context the distance units have smaller vertical than horizontal extent, with the result that vertical assessments are more precise than horizontal assessments.

Finally, if we grant that the size assessment is related to separation assessment, we find that certain peripheral aspects of size assessment are consistent with the indirect view. An object is perceived as larger when its image falls on a compressed retina and smaller when its image falls on a stretched retina (Duke-Elder, 1934; Critchley, 1953; Winn et al., 1988). Further, an object is perceived as smaller when it is observed peripherally than when it is observed more foveally (Newsome, 1972; Schneider et al., 1978; Thompson and Fowler, 1980).

## Brain bases

What are the brain bases of the indirect separation assessment process that Hisakata et al. (2016), Song et al. (2017), Dopkins and Galyer (2020), and Dopkins and McIntire (2022) propose? The distance units are probably represented early in the visual system. Consistent with this view, V1 has been linked with judgments of Vernier acuity, which have been localized at the same level of processing as judgments of separation (Duncan and Boynton, 2003; Vesker and Wilson, 2013). Similarly, V1 has been linked with judgments of size, which are related to judgments of separation. The critical results were observed under conditions in which two stimuli encompassed the same visual angle, one stimulus seemed farther away than the other, and the far stimulus was perceived as being larger than the near stimulus (e.g., in the Ponzo illusion). In this situation, the far stimulus, which was perceived as larger, activated a larger area in V1 than the near stimulus, which was perceived as smaller (Murray et al., 2006; Fang et al., 2008; see also Pooresmaeili et al., 2013). Thus, perceived size was positively correlated with the amount of V1 activated. As a possible account of these results MacEvoy and Fitzpatrick (2006) proposed that, in this situation, neurons in V1 shift their receptive fields toward the center of the stimulus so that the boundaries of the stimulus encompass a larger number of neurons. Under this account the shift has a functional basis in that the perceived size of a stimulus depends on the number of V1 neurons involved in representing the stimulus. Neurophysiological and fMRI evidence supports the claim of receptive field shift. Early reports suggested that the diameters of some human and monkey V1 receptive fields increase with decreases in viewing distance (Marg and Adams, 1970; Smith and Marg, 1975). Later reports suggested that receptive fields are tuned to the ‘perceived’ as opposed to the retinal size of the stimuli (Ni et al., 2014; He et al., 2015). More generally, judged size is correlated with V1 population receptive field size (Moutsiana et al., 2016). On the basis of this research V1 has been accorded an important role in judgments of size. Notice also that the forgoing results are consistent with the indirect account of separation assessment in that they suggest a relationship between perceived size and the amount of V1 activation.

Other results support the account further. The magnitude of several size illusions is negatively correlated with surface area of V1 (Schwarzkopf et al., 2011; Schwarzkopf and Rees, 2013). These results suggest the relevance of lateral V1 connections for size judgments because the impact of these connections is reduced when V1 surface area is larger (Schwarzkopf, 2015). Such lateral connections have been proposed as a mechanism for the integration of distance units (Song et al., 2017). Finally, V1 has been said to have the structure of a scale-space, comprising fields of Gaussian ‘samplers,’ of differing grains, with samplers at more refined grains being sampled by samplers of coarser grains, such that samplers of all grains at a given spatial location are correlated (Koenderink, 2019). Such a multi-scale representation is consistent with proposals that the distance units underlying separation assessment can vary in size (Dopkins and Galyer, 2020; Dopkins and McIntire, 2022).

It makes sense that V1 would be involved in separation and size assessment. First, visual representation is more refined here than elsewhere in the brain (Srinivasan et al., 2015). Second, V1 represents visual space very consistently. The cortical magnification factor (CMF), the number of neurons dedicated to processing a part of the visual field, decreases with increases in distance from the center of the visual field (eccentricity). At the same time the size of the population receptive field (pRF) increases with eccentricity. As a result of the aforementioned two relationships, the population point image size, the product of CMP and pRF size, is nearly constant as a function of eccentricity. By implication, the point image size may be constant as a function of eccentricity (Harvey and Dumoulin, 2011).

Of course, other brain areas in addition to V1 are presumably involved in separation and size assessment. Mechanisms that integrate distance units are probably housed later in the system, possibly serving multiple modalities (Hisakata and Kaneko, 2021). TMS, lesion, and imaging results suggest that the contextual information (e.g., depth information) needed for size scaling is incorporated by higher visual areas such as the lateral occipital cortex (Frassinetti et al., 1999; Servos, 2006; Plewan et al., 2012; Zeng et al., 2020). Imaging data imply that the right superior parietal cortex supports the actual evaluation process (Plewan et al., 2012). Explicit values of separation and size may even be represented cortically. An area of the parietal cortex has been reported to support topographically organized tuning for visual object size (Harvey et al., 2015).

Of relevance to the brain bases of separation and size assessment are proposals that length, size, duration, numerosity are assessed in terms of a generalized magnitude representation (Dormal and Pesenti, 2007; Walsh, 2003; Lourenco and Longo, 2011; Bonn and Cantlon, 2017; Martin et al., 2017). Magnitude assessment in terms of this representation is often conceived in Bayesian terms as process of accumulation (Martin et al., 2017). Supporting this view are studies showing involvement of overlapping parietal areas with magnitude judgments of various sorts (Bueti and Walsh, 2009; Dormal and Pesenti, 2009, 2012; Dormal et al., 2012; Cona et al., 2021) and behavioral studies showing cross-dimensional interactions in magnitude judgment (Lambrechts et al., 2013). The view of separation as being assessed in terms of a generalized magnitude representation comports with the indirect view of separation assessment.

## Summary

In sum, recent results support a view under which visual separation is assessed through the integration of distance units. These units may be housed in V1, with integrative processes occurring in later areas of the visual system. Little support has emerged for the rival view under which visual separation is assessed through the comparison of position values. These conclusions regarding visual separation are paralleled by similar conclusions regarding the somatosensory system. Here, position is apparently coded in population terms, with the differences in the profile of neural activity across fibers in a nerve increasing with the distance between the pairs of positions stimulated (Ray and Doetsch, 1990). Brushing the skin produces patterns of activity that are distributed across surprisingly large regions of the cortex (Tommerdahl et al., 1993). Tactile separation is said to be assessed by ‘counting’ the number of neurons between the somatotopic map locations for the points at which stimulation is occurring (Longo, 2006). Distortions and anisotropies in the assessment of tactile separation have been explained in terms of this account.

## Data availability statement

The original contributions presented in the study are included in the article/supplementary material, further inquiries can be directed to the corresponding author.

## Author contributions

SD: Conceptualization, Data curation, Formal analysis, Funding acquisition, Investigation, Methodology, Project administration, Resources, Software, Supervision, Validation, Visualization, Writing – original draft, Writing – review & editing.
